# The Urine in Skin Diseases

**Published:** 1914-12

**Authors:** H. C. Baum

**Affiliations:** Syracuse, N. Y.; 809 University Block


### BUFFALO MEDICAL JOURNAL

Yearly Volume 70. DECEMBER, 1914 No. 5

### ORIGINAL ARTICLES

The right is reserved to decline papers not dealing with practical medical and surgical subjects, and such as might offend or fail to interest readers. Contributors are solely responsible for opinions, methods of expression and revision of proof.

### The Urine In Skin Diseases.

By H. C. BAUM, M. D., Syracuse, N. Y.

The skin reacts variably to irritation. This irritation may be from without or from within. A wide range of reaction is seen in different individuals and in the same individuals at different periods. This variation has been explained as resulting from varying susceptibility of the skin. What constitutes this susceptibility? Our medical ancestors were wont to attribute it to "Humors." And the treatment they employed was not very different from that employed at the present time. But neither the modern patient nor the modern physician is satisfied with the vagueness of the theory and especially since 1879 serious work has been done with the aim of throwing light on some of the dark places in etiology.

No doubt the work of Wright has been, in its sphere, as prolific of practical advance as any. Vaughn has led investigation along other lines. American physiological chemists are accomplishing much in unraveling the mysteries of metabolism and it seems not unreasonable to suppose that another generation of medical men will have the advantage of a relatively clear view of the chemistry of health and disease.

Contributors to the advance in medicine are not all from the ranks of laboratory men. Clinicians always have and always will contribute their part. It is a mistake to suppose that the clinician may also be the laboratory man, save in a limited and unsatisfactory degree. The busy general practitioner must satisfy himself with simple laboratory efforts and depend on the laboratory man to supply the technical and exacting methods of analysis. But well within the scope of the general practitioner and the majority of specialists are the simple and very practical examinations that surely constitute the preponderance of laboratory work essential to the proper conduct of treatment in most cases.

Clinical study of a large number of cases of skin disease, of which little is known of that phase of the etiology previously referred to as susceptibility of the skin, has impressed me with the significance of certain findings in the urine in two common types of skin disease. And it is for the purpose of attracting your attention to these findings and the aid they give in planning treatment that these notes are offered.

In diseases of the sebaceous gland system, it is common to find indican or one of the kindred toxic bodies present in excess in the urine. In this group are the familiar cases of excessive oiliness of the scalp and skin where it is rich in sebaceous glands. In acne vulgaris, comedo, seborrheic dermatitis, seborrhoea capitis (with or without alopecia) and even in the overgrowth of hair on the faces of women, it is commonly found.

Pusey states that the sweat glands may eliminate indican. But it is not my experience to find diseases of the sweat glands commonly associated with indican excess. Where this is found there is usually also an acidosis, and it is with the latter, not to the indican, that this type of lesion should be associated. It has long seemed to me that in no other way can this association be explained than by assumption that the sebaceous glands eliminate indican or some products associated, are irritated, and thereby rendered susceptible to disease.

Diseases of the sebaceous gland system are apt to be both stubborn and recurrent. If one may detect at least a part of the etiologic susceptibility, it ought to go far in getting better results than by the old routines.

A second type of skin diseases, as already hinted, seem to be associated with acidosis. Certain erythemas, the explosive erythematous eczemas, so often mistaken for erysipilas, the acute eczemas of vesicular type and some of the chronic type of so-called eczemas are well known to be associated with acidity. There are forms associated with the rheumatic diathesis and which yield promptly to accurate antirheumatic regimen, diet and medication, even without local treatment.

A habit of studying the urine of nearly all skin cases had led to these convictions.

As a result, constitutional treatment has usually been modified according to the evidence furnished by the urine. It has led to a study of the elimination of those contributing states, both the correction of the intoxication and the formation of the intoxicating substances. One is impressed with the dearth of helpful literature and by the difficulty of wringing a confession of conviction from other medical men as to a dependable treatment for either the acid diathesis or the indican intoxication.

There is an acute and a chronic autointoxication. The ephemeral type is not capable of inducing skin disease that is troublesome, for the reason that the symptoms, if any, disappear with the cause. But in the chronic, ingrained toxaemia, of either type, the practitioner is put to his trumps to succeed in effecting a permanent betterment.

The oxaluria associated with chronic and severe skin diseases is believed by Vaughn to be the effect of loss of skin function and not a cause of the skin lesions. I have not been able to determine any definite type of skin lesions associated with oxaluria.

No promise is made, in the title of this paper, of any remarks on treatment. And the writer has no convictions, even concerning general regimen, which he is not willing to amend. With our inexact knowledge of metabolic processes and the sources of their derangement we are left to imperic treatment. In this connection it is interesting to recall the work of Boeck, nearly a half century ago. He assumed that lichen planus resulted from the absorption, from the intestines, of a putrefactive humor, and that with more oxygen, the system would not develope the putrefactive bodies. As in the laboratory oxygen could be made from the reaction of nitric acid on chlorate of potassium, he gave chlorate of potassium immediately after meals and nitric acid, well diluted, half an hour or so later. The lichen planus disappeared. His plan of treatment was accepted very generally. Such men as L. Duncan Bulkley and the late R. W. Taylor, have repeatedlv extolled the "almost marvelous effect of this treatment in lichen planus, without committing themselves to the underlying theory. They said they did not know how it acted, but it seemed their first and best recourse in this severe and often intractible disease.

Wishing to test, as far as possible, Boeck's theory, the treatment has been employed in a very large group of cases exhibiting suboxide toxaemia, especially of the indican type. The prompt clearing up of the urine has led me to believe that he was quite right. And incidentally, the skin disease, often of a sluggish, unresponsive character, proves more amenable to the remedies employed.

Just how the Boeck treatment acts is still debatable. It is not my desire to offer any theory to explain it. Chlorate of potassium alone will not accomplish the same result, nor will nitric acid. The temporary clearing up of the indican does not accomplish all that we wish, but is certainly breaks the vicious circle and enables the system to proceed more normally in its functions. Not infrequently the treatment is again indicated after a few weeks or months. In others indican does not tend to recur, especially if the patient adopts measures to deter it.

Neither for the condition for which I am in the habit of employing the term acidosis, nor for that of indican intoxication have I found any treatment uniformly successful and satisfactory. In these cases many influences are at work. Obscure nervous elements, wrong environment, bad hygiene and habits and many other influences may play a part. Remote infections, from decayed teeth, diseased tonsils, etc., seem contributory. And at present it is thought that the internal secretion may become disturbed and that metabolic changes result. Defective elimination, which is meant to include retention of nitrogen and other effete substances, probably has a large role in the etiology of skin diseases, but whether this is a primary disease condition is at least open to doubt.

809 University Block.

---

**A New Reaction For Syphilis.**—Landau (Wien. klin. Wochenschr., No. 42, 1913). The reagent consists of 5 drops of tincture of iodine in 50 c.cm. white paraffine oil. This mixture must always be freshly prepared. The indicator is a clear solution of boiled potato starch. Of the blood-serum, obtained in the usual manner, 0.2 c.cm. are placed in a narrow test-tube, 2.5 c.cm. of the reagent are added and the mixture thoroughly shaken until nearly decolorized. The tube is then closed with a rubber stopper and left lying flat, in a dark place, at room temperature, for two to four hours, after which a few drops of the starch solution are added. Normal sera take on a deep blue color; syphilitic sera remain unchanged. The reaction is apparently due to the presence in syphilitic serum of substances, perhaps fatty acids, that have a strong affinity for iodine, but only in the presence of a lipoid like paraffine. In 77 cases of syphilis, of all kinds, the iodine reaction was positive in 68, the Wasserman test in only 47. All non-syphilitic cases were negative with both tests.

---

**Dressings With Horse-Serum in Pediatrics** and Especially in burns. P. Barlerin, "Bulletin Mensuel de la Société d'Hygiene de l'Enfance," June, 1914, lauds the effect of dried horse-serum in local applications. He claims that it produces a localized leucocytosis and that it acts as a disinfectant and microbicide through phagocytosis as well as a regenerative and cicatrisator. He describes two cases of burns treated with this method and also recommends it in other conditions such as angina, amygdalitis, abscesses, phlegmons, osteomyelitis, impetigo and also as a hemostatic.—C. G. L-W.

